# Self-reported food intolerance, dietary supplement use and malnutrition in chronic inflammatory bowel diseases: Findings from a cross-sectional study in Lebanon

**DOI:** 10.1371/journal.pone.0305352

**Published:** 2024-07-25

**Authors:** Maha Hoteit, Nour Ftouni, Malak Olayan, Souheil Hallit, Joya Maria Karam, Mahmoud Hallal, Samer Hotayt, Bilal Hotayt

**Affiliations:** 1 Food Science Unit, National Council for Scientific Research-Lebanon (CNRS-Lebanon), Beirut, Lebanon; 2 Faculty of Public Health, Lebanese University, Beirut, Lebanon; 3 School of Medicine and Medical Sciences, Holy Spirit University of Kaslik, Jounieh, Lebanon; 4 Applied Science Research Center, Applied Science Private University, Amman, Jordan; 5 Faculty of Medicine, Lebanese University, Beirut, Lebanon; 6 Gastroenterology Department, Faculty of Medical Science, Lebanese University, Beirut, Lebanon; 7 Gastroenterology and Hepatology Department, Zahraa University Medical Center (ZHUMC), Beirut, Lebanon; 8 Anesthesia Department, Saint Joseph Hospital, Paris, France; 9 Gastroenterology Department, Sahel General Hospital, Beirut, Lebanon; Fonterra Coop / Lebanese University, LEBANON

## Abstract

**Background/Aims:**

Chronic inflammatory bowel diseases (IBDs), including Crohn’s disease and ulcerative colitis are known for a combination of food intolerance, decreased oral intake, and malabsorption which all predispose patients to malnutrition and suboptimal dietary intake. The present study was conducted to 1) examine self-reported food intolerances and dietary supplement use 2) assess nutritional intake 3) assess the nutritional status and screen for malnutrition among patients with chronic inflammatory bowel disease (CIBD).

**Methods:**

48 patients with CIBDs (28 Crohn’s disease, 15 ulcerative colitis and 7 with atypical forms of IBD) took part in this cross-sectional study. Participants completed a food frequency questionnaire targeting dietary intakes and food trends over time. A questionnaire about food intolerance was also used. The nutritional status of patients with CIBDs was evaluated by a detailed history (medical diagnosis and medications and supplements administered) and by using the subjective global assessment (SGA) tool. Anthropometric data including height, weight, and BMI with body composition assessment using automated scales and stadiometer, while Bio-impedancemetry was used to measure body fat and visceral fat. Statistical analysis was conducted using SPSS 27, employing mean values, standard deviations, absolute and relative frequencies and Pearson’s chi-square test, with significance set at p ≤ 0.05.

**Results:**

Food intolerance was equally common in all the types of CIBD specifically for dairy products, spicy foods, and high-fiber food items (beans and raw vegetables). Individuals with CIBD were also complaining about meat and chicken products (68%), followed by alcohol and soda (64%) and fish and sea foods (59%). 17% of the patients were malnourished. A significant percentage of malnourished patients with CIBD had to follow a diet outside the flare, had a nutritional follow up, were currently taking corticosteroids and had a severe form of the disease compared to patients who were well nourished.

**Conclusions:**

This study has contributed valuable insights into the understanding that some food items could be associated to periods of increased disease activity in CIBD patients and that awareness/intervention regarding nutrition must be provided by healthcare professionals (dietitians, physicians…) to decrease the need for second line therapy. In addition, this self-reported food intolerance paper gives an insight for patients on food items usually avoided by CIBD patients during flares.

## Introduction

Chronic inflammatory bowel disease (CIBD) is defined as a non-infectious inflammatory condition that affects the colon and small intestine in genetically susceptible individuals [1]. It primarily includes Ulcerative Colitis (UC), Crohn’s disease (CD) and Intermediate Colitis (IC) [2]. According to data collected from 195 countries spanning from 1990 to 2017, the global prevalence of CIBD surged [3] from 3.7 million (95% UI 3.5–3.9) to more than 6.8 million (6.4–7.3), reflecting a rise of 85.1% (79.5–89.9). Similarly, a heightened incidence of CIBD has been reported in the Arab world particularly among males [4]. In Lebanon, a novel study revealed that the annual incidence of CIBD was 5.5 per 100,000 people per year with an annual incidence of 4.1 per 100,000 people for UC and 1.4 per 100,000 people for CD [4]. A larger number of existing studies in broader literature have examined risk factors of CIBD, for instance genetics and environment. Dietary habits play a homeostasis role in the gut’s microbial composition and functioning. The composition of the ingested foods modulates mucosal barrier function, gut immunity, and gut inflammation [5]. Nutritional assessment is a key aspect in controlling CIBD [6]. By tracking food intake, patients with CIBD might link their gastrointestinal symptoms to the consumption of certain food items, pushing them to implement dietary restrictions for fear of triggering or exacerbating a pre-existing flare, with consequent decrease in nutritional intake [7]. This can impact the nutritional quality of their diet resulting in micronutrient deficiency as well as obesity [8]. For instance, foods that have been most reported to cause CIBD symptoms were cereals and milk products. Also, most vegetables, being more frequently blamed than other food components, except for onions and cabbage, cause gastrointestinal discomfort [7]. Furtherly, according to MacDermott [9], the explicit categories of foods and beverages with the capacity to trigger gastro-intestinal symptoms consist of caffeine-containing items, alcoholic drinks, fruits, spices, flavorings, condiments, fast-food items, as well as cookies, crackers, pretzels, cakes, and other. Conversely, there are varieties of foods and drinks that may be more easily tolerated, such as water, rice, simple pasta, or noodles, baked or boiled potatoes, white bread, plain fish, chicken, turkey, ham, eggs, and others. Moreover, it has been reviewed that patients with UC and CD share a similar occurrence and pattern of food intolerance owing to the activity of the disease, its location, or a past surgical intervention [7]. This implies that food intolerance is related to the condition of each patient and accordingly, treatment must also be administered with a specific approach for each person.

Malnutrition and nutritional deficiencies are commonly found in CIBD. Malnutrition occurs with frequency in CD in contrast with UC and this has been linked to the inflammation affecting the small intestine in CD [6]. According to many solid-based evidence, malnutrition rate can reach 30% to 75% of patients with CD and 18% to 62% of patients with UC [10–12]. Malnutrition is due to the nutrient losses due to symptoms such as diarrhea and to pathophysiological causes such as the inflammation of the mucosal membrane [6]. The severity and the extent and duration of disease modulate the magnitude of malnutrition among patients with CIBD; however, even patients in remission can suffer from malnutrition which is associated with worsening of the symptoms, health-related complications and death [2]. As part of comprehensive CIBD care, it is important for clinicians and dietitians to assess the nutritional status, and this would include measuring body composition, controlling dietary intake and supplement use as well as make a solid diagnosis to intervene and monitor patients with CIBD. The role of gut microbiota and vitamins in CIBD has garnered significant attention in recent years due to their crucial impact on disease development and progression. The gut microbiota, a complex community of microorganisms residing in the intestines, plays a vital role in maintaining gut health and immune function. Imbalances in the gut microbiota, known as dysbiosis, have been linked to the pathogenesis of CIBD. Probiotics, prebiotics, and dietary interventions aimed at modulating the gut microbiota composition have shown promise in managing CIBD symptoms and reducing inflammation [13]. Additionally, vitamins such as vitamin D, vitamin B12, and folate play essential roles in immune regulation and gut health. Deficiencies in these vitamins have been associated with increased disease severity and risk of flare-ups in CIBD patients [14]. Therefore, optimizing gut microbiota composition and ensuring adequate vitamin intake are crucial aspects of managing and potentially preventing inflammatory bowel disease. Alongside medical treatment regimens, enhancing nutrition and dietary habits have been the primary concern of clinicians and dietitians in order to help patients alleviate their symptoms and improving overall well-being [15, 16]. However, the findings about self-reported supplementation are multifaceted and often poorly understood. Despite the increase consumption and awareness regarding the importance of dietary supplementation and vitamins [15, 17, 18], the efficacy and benefits in the context of CIBD remains unclear. That’s why understanding the patterns and motivations behind supplement consumption amid and outside CIBD flare is crucial for more evidence- based guidance to optimize patient care. As far as we know, no previous research in Lebanon has investigated the occurrence of malnutrition and the nutritional status among CIBD diagnosed individuals. Understanding the specific patterns and the challenges of dietary intake faced by patients with CIBD is essential to ensure successful management. A recent study conducted in Lebanon studied the progression of CIBD during a period of 20 years. In fact, a progressive increase of incidence has been reported among the sample, especially for CD patients. In Lebanon, the prevalence of UC and CD were 69.9 per 100,000 and 68.35 per 100,000 respectively [19]. Therefore, the increasing prevalence of CIBD in Lebanon prompts us to study the patients’ food behaviors during or out flare. Our study aims to determine the frequency and types of food that CIBD patients perceive as intolerable or triggering symptoms which would provide an insight into dietary challenges faced by those patients. Moreover, nutritional imbalances and risk of malnutrition were also assessed.

## Methods

### Ethical approval

The Lebanese University ethical committee approved the study protocol (#153/Dec 5, 2014). Before the study commenced, each participant signed an informed consent. It is important to note that the consent form was secured for all participants.

### Description of participants

Our team accessed the medical records at Al-Sahel General Hospital-Mount-Lebanon from which the list of patients’ contact information was retrieved for the years 2015 and 2016. This retrospective inspection occurred between February and April 2017. A total of 76 patients with CIBD were contacted. The response rate was 67%. Out of 76 participants, 48 patients were enrolled, and they aged 18 years old and above (19 men and 29 women) with the diagnosis of either CD, UC, or IC (28 CD, 15 UC and 7 IC). Patients were contacted to come for a scheduled clinic appointment at the Lebanese university medical center. The sample size was driven by the number of patients who consented to participate, rather than through sample size/power calculations. Inclusion criteria comprised individuals aged above 18 years, those who signed the informed consent, and had a confirmed CIBD diagnosis. Participants that lacked confirmed diagnosis or had any element that might influence the nutrition status such as pregnancy, history of cancer, eating disorders, renal diseases, etc. were disqualified from participation.

### Instruments

All the patients were face-to-face interviewed by trained dietitians to fill a questionnaire that encompasses socio-demographic, economic and health-related features. Additionally, the patients were requested to complete a survey to assess the CIBD type; and further information have been collected such as disease diagnosis year, location of the affected section in the digestive tract, surgical or fistula-related history as well as the use of medications and dietary supplements. The completed medical questionnaire related to the patients with CIBD was compared and verified using the hospital chart reviews. The Harvey-Bradshaw index was employed to assess disease activity in Crohn’s disease (CD) patients, while the Powell-Tuck Index was utilized for those with ulcerative colitis (UC) [20].

### Food frequency questionnaire

The trained dietitian completed with each patient a quantitative food frequency questionnaire, consisting of 164 food items with serving size and frequency of consumption for each item.

The target of this questionnaire is to assess dietary patterns, identify food intake trends over time and evaluate nutrient intake. Prior to use, the questionnaire was validated to ensure its reliability and accuracy in the assessment of dietary habits [21].

Subjects were given a form to track all foods consumed during 3 successive days, one of which being a weekend day. Before the start of the 3 days’ food record, patients were given clear verbal and written guidelines. Participants were inquired about the type and frequency of all their dietary supplement usage. They were also instructed to bring along these supplements for the dietitian to examine.

Nutrient intake was assessed by using Nutritionist Pro software (version 3.2, AXXYA). Local foods were available at the time of analysis in this software. The mean and standard deviation (SD) were employed for interpreting nutrient intake. Insufficient nutrient intake was denoted by a level falling below the recommended Dietary Reference Intake (DRI) [17, 18].

### Subjective Global Assessment (SGA)

A method utilized to help in the diagnosis of malnutrition [19]. This assessment tool includes tracking recent food consumption, alterations in weight, gastrointestinal symptoms, and a clinical assessment. The SGA tool is valid for application among patients with CIBD [19]. SGA ratings were characterized as: A: Normal/well nourished; B: Mildly/Moderately malnourished; C: Severely malnourished.

### Food intolerance questionnaire

The food intolerance questionnaire is a self-reported questionnaire designed to assess tolerance of 113 food items for patients having CD or UC. The patients provided one of three responses for each food item: tolerance, intolerance, or dislike. [7].

### Anthropometric measures

Nutritional status assessment relied on observing anthropometric data, encompassing weight, height, and body mass index (BMI). An automated scale and stadiometer were used to measure participant weight and height. BMI was computed using the standard formula, dividing weight in kilograms (kg) by the square of height in meters (m^2). Furthermore, body composition, involving body fat, lean body mass, and visceral fat, was evaluated using a Bio-impedancemetry body composition analyzer (BF-511, OMRON®). Percentage body fat (%BF) was determined through whole-body BIA using a digital scale/body composition monitor (Omron BF 511, 50 kHz, 500 μA, Kyoto, Japan) and was rounded to the nearest 0.1%. To categorize study subjects into groups of low, normal, high, and very high percentage of body fat (%BF), the Omron Healthcare standards based on age and gender were employed.

### Statistical analyses

The research data was organized using Microsoft Excel® and Word® software. Statistical analysis was conducted using SPSS 27 software. Mean values and standard deviations were employed for quantitative variables, while absolute and relative frequencies described categorical variables. Pearson’s chi-square test was utilized to study associations among categorical variables. Differences were deemed significant if the p-value was 0.05 or lower (p ≤ 0.05).

## Results

Our results showed that 60% percent are females. More than 50% of the participants had CD, aged less than 35 years old and were married with no children. Three quarters of the study participants were educated and currently working. More than seventy percent of them were non-smokers, consumed their daily meals irregularly and presented a sedentary lifestyle. None of them consumed alcohol (Table 1). The mean BMI was 26.30 ± 5.28 kg/m^2^. All details about the treatments of the participants and their nutritional status are included in Table 2, dietary supplements in Table 3, characteristics of dietary patterns, nutrition advice and sources of dietetic information amid and outside flares in Table 4. One in four participants were obese, with 73% having high fat mass and 36% having low muscle mass (Table 2). Based on SGA, 17% of participants were at risk of mild to severe malnutrition (Table 2). As for dietary supplements, 85% of participants were not taking any supplements either amid flares, or during remission episode (Table 3). As for the dietary patterns of patients with IBD, the percentage of participants adhering to diet prescription decreases from 50% amid flares to 25% during remission period. Amid flares, few people stop eating fruits and vegetables (20.8%), peeled their fruits (29.2%), stop eating fried foods (31.3%), stop eating salty foods and snacks (16.7%) and stop eating sugary foods and snacks (12.5%). This prevalence decreases more in the remission period (see Table 4). As for dietary advice sources, only 2% of participants were referred to dietitians amid flares. During the remission period, 75% of patients with IBD don’t follow-up anymore with their physicians or dietitians. As for the nutritional follow-up, only 10% reported visiting dietitians in their clinics (Table 4). The assessment of patient’s dietary intake shows that most patients presented deficiencies in all the vitamins listed in Table 5, except for vitamin K (p = 0.04) and phosphorous (p = 0.03).

**Table 1:** Socioeconomic characteristics of the sample.

Variables	
	Mean ± SD
**Age**	36.79 ± 13.06
	**N (%)**
**Age categories**	
18–24	12 (25)
25–64	34 (70.8)
65+	2 (4.2)
Total	48 (100)
**Sex**	
Male	19 (39.6)
Female	29 (60.4)
**Tobacco**	
No	34 (70.8)
Yes	14 (29.2)
**Alcohol**	
No	48 (100)
Yes	0 (0)
**Sports**	
No	38 (79.2)
Yes	10 (20.8)
**Profession**	
Currently working	15 (31.3)
Currently unemployed	33 (68.8)

**Table 2:** Anthropometric data and medical characteristics of study population.

Variables	N (%)
**Type of CIBD**	
CD (Crohn’s disease)	27 (56.3)
UC (Ulcerative Colitis)	15 (31.3)
IC (Indeterminate colitis)	6 (12.5)
**Clinical activity**	
Mild activity	29 (60.4)
Moderate activity	8 (16.7)
Severe activity	11 (22.9)
**Number of flares per year**	
0–12	44 (91.7)
13–24	2 (4.2)
25–36	0 (0)
36 and above	2 (4.2)
**Number of hospitalization due to flares per year**	
0	37 (77.1)
1	5 (10.4)
2	4 (8.3)
3+	2 (4.2)
**Type and mode of treatments**	
**5-ASA (Aminosalicylic acids)**	
No	32 (66.7)
Yes	16 (33.3)
**If Yes: mode of treatment**	
Enemas	2 (4.2)
Oral	14 (29.2)
**Corticosteroids**	
No	36 (75)
Yes	12 (25)
**Colofoam**	
No	48 (100)
**Immunosuppressants**	
No	48 (100)
**Biological treatment**	
No	40 (83.3)
Yes	8 (16.7)
**Surgical history**	
No	46 (95.8)
Yes	2 (4.2)
**Number of surgeries**	
0	46 (95.8)
1	2 (4.2)
**Surgery type**	
No previous surgeries	46 (95.8)
Ablation small intestine	1 (2.1)
Ablation small intestine and colon	1 (2.1)
**Anthropometry measurements**	
**Weight (kg)**	75.75±18.09
**Height (cm)**	167.91±9.44
**Body Mass Index (kg/m** ^**2**^ **)**	26.30 ± 5.28
**Waist circumference (cm)**	88.63 ± 20.61
**Body Mass Index (BMI) categories**	
Underweight	2 (5.3)
Normal body weight	15 (39.5)
Overweight	12 (31.6)
Obesity	9 (23.7)
**Fat mass (kg)**	34.85 ± 10.19
**Fat mass categories**	
Low	1 (3)
Normal	4 (12.1)
High	4 (12.1)
Very high	24 (72.7)
**Visceral mass (kg)**	7.64 ± 4.27
**Visceral fat categories**	
Normal	21 (63.6)
High	10 (30.3)
Very high	2 (6.1)
**Muscle mass (kg)**	28.94 ± 6.43
**Muscle mass categories**	
Low	12 (36.4)
Normal	10 (30.3)
High	4 (12.1)
Very high	7 (21.2)
Malnutrition according to SGA* **rating**	
Well nourished	40 (83.3)
Mild-moderate undernutrition	5 (10.4)
Severe undernutrition	3 (6.3)

*SGA: Subjective global assessment

**Table 3:** Dietary supplements use by patients with CIBD.

	N (%)
**Dietary supplements**	
No	41 (85.4)
Yes	7 (14.6)
**Vitamins**	
None	43 (89.6)
Vit D	4 (8.3)
Vit E	1 (2.1)
**Trace elements**	
None	46 (95.8)
Fe	2 (4.2)
**Oral liquid supplements**	
No	48 (100)
**Probiotics**	
No	48 (100)
**Another type of supplements**	
No	48 (100)
**Sources of dietary supplements prescription**	
No one	41 (85.4)
Physician	6 (12.5)
Dietitian	1 (2.1)
**Dietary supplements use amid flares**	
No	44 (91.7)
Yes	4 (8.3)
**Dietary supplements use in the remission episode**	
No	48 (100)

**Table 4:** Characteristics of dietary intake, nutrition advice and sources of dietetic information amid and outside flares among patients with CIBD.

Variables	N (%)
**Adherence to diet prescription amid flares**	
No	24 (50)
Yes	24 (50)
**Stop eating fruits and vegetables**	
No	38 (79.2)
Yes	10 (20.8)
**Eating peeled fruits and vegetables**	
No	34 (70.8)
Yes	14 (29.2)
**Stop eating fried foods**	
No	33 (68.8)
Yes	15 (31.3)
**Stop eating salty foods and snacks**	
No	40 (83.3)
Yes	8 (16.7)
**Stop eating fatty foods and snacks**	
No	36 (75)
Yes	12 (25)
**Stop eating sugary foods and snacks**	
No	42 (87.5)
Yes	6 (12.5)
**Sources of dietary advices amid flares**	
No one	24 (50)
Physician	16 (33.3)
Dietitian	1 (2.1)
Personal belief	7 (14.6)
**Adherence to diet prescription in the remission episode**	
No	36 (75)
Yes	12 (25)
**Stop eating fruits and vegetables**	
No	46 (95.8)
Yes	2 (4.2)
**Eating peeled fruits and vegetables**	
No	47 (97.9)
Yes	1 (2.1)
**Stop eating fried foods**	
No	43 (89.6)
Yes	5 (10.4)
**Stop eating salty foods and snacks**	
No	43 (89.6)
Yes	5 (10.4)
**Stop eating fatty foods and snacks**	
No	47 (97.9)
Yes	1 (2.1)
**Stop eating sugary foods and snacks**	
No	43 (89.6)
Yes	5 (10.4)
**Sources of dietary advice outside flares**	
No one	36 (75)
Physician	8 (16.7)
Personal belief	4 (8.3)
**Soft drink consumption amid and outside flares**	
No	20 (41.7)
Yes	28 (58.3)
**Type**	
Sweet	26 (54.2)
No sugar/sweetened	2 (4.2)
None	20 (41.7)
**Origin of water**	
Water bottle	46 (95.8)
Tap water	2 (4.2)
**Type of water**	
Mineral water	48 (100)
**Nutritional follow up in nutrition clinics**	
No	43 (89.6)
Yes	5 (10.4)
**Nutritional follow up with healthcare professionals**	
No one	43 (89.6)
Physician	2 (4.2)
Dietitian	3 (6.3)

**Table 5:** Micronutrients intake and corresponding benchmarks based on the recommended daily allowances (RDA)* of CIBD diagnosed patients by age and gender.

		Youth					Adults					Seniors				
		sex				p-value	sex				p-value	sex				p-value
		men		women			men		women			men		women		
		N	%	N	%		N	%	N	%		N	%	N	%	
Vit A	deficient	5	100.0%	6	85.7%	0.37	12	85.7%	17	85.0%	0.95	0	0.0%	2	100.0%	-
	normal	0	0.0%	1	14.3%		2	14.3%	3	15.0%		0	0.0%	0	0.0%	
Vit D	deficient	5	100.0%	7	100.0%	-	14	100.0%	20	100.0%	-	0	0.0%	2	100.0%	-
	normal	0	0.0%	0	0.0%		0	0.0%	0	0.0%		0	0.0%	0	0.0%	
Vit E	deficient	4	80.0%	7	100.0%	0.21	12	85.7%	20	100.0%	0.08	0	0.0%	2	100.0%	-
	normal	1	20.0%	0	0.0%		2	14.3%	0	0.0%		0	0.0%	0	0.0%	
Vit K*	deficient	5	100.0%	7	100.0%	-	14	100.0%	15	75.0%	0.04	0	0.0%	2	100.0%	-
	normal	0	0.0%	0	0.0%		0	0.0%	5	25.0%		0	0.0%	0	0.0%	
Vit B3	deficient	2	40.0%	3	42.9%	0.92	6	42.9%	14	70.0%	0.11	0	0.0%	1	50.0%	-
	normal	3	60.0%	4	57.1%		8	57.1%	6	30.0%		0	0.0%	1	50.0%	
Vit B12	deficient	4	80.0%	7	100.0%	0.21	9	64.3%	18	90.0%	0.06	0	0.0%	2	100.0%	-
	normal	1	20.0%	0	0.0%		5	35.7%	2	10.0%		0	0.0%	0	0.0%	
Vit C	deficient	5	100.0%	6	85.7%	0.37	13	92.9%	17	85.0%	0.48	0	0.0%	2	100.0%	-
	normal	0	0.0%	1	14.3%		1	7.1%	3	15.0%		0	0.0%	0	0.0%	
Copper	deficient	5	100.0%	7	100.0%	-	14	100.0%	20	100.0%	-	0	0.0%	2	100.0%	-
	normal	0	0.0%	0	0.0%		0	0.0%	0	0.0%		0	0.0%	0	0.0%	
Phosphorus	deficient	5	100.0%	7	100.0%	-	11	78.6%	20	100.0%	0.03	0	0.0%	2	100.0%	-
	normal	0	0.0%	0	0.0%		3	21.4%	0	0.0%		0	0.0%	0	0.0%	
Magnesium	deficient	5	100.0%	7	100.0%	-	13	92.9%	20	100.0%	0.22	0	0.0%	2	100.0%	-
	normal	0	0.0%	0	0.0%		1	7.1%	0	0.0%		0	0.0%	0	0.0%	
Zinc	deficient	5	100.0%	7	100.0%	-	14	100.0%	20	100.0%	-	0	0.0%	2	100.0%	-
	normal	0	0.0%	0	0.0%		0	0.0%	0	0.0%		0	0.0%	0	0.0%	
Selenium	deficient	4	80.0%	2	28.6%	0.07	8	57.1%	14	70.0%	0.44	0	0.0%	1	50.0%	-
	normal	1	20.0%	5	71.4%		6	42.9%	6	30.0%		0	0.0%	1	50.0%	
Iron	deficient	4	80.0%	6	85.7%	0.79	11	78.6%	18	90.0%	0.35	0	0.0%	2	100.0%	-
	normal	1	20.0%	1	14.3%		3	21.4%	2	10.0%		0	0.0%	0	0.0%	
Calcium	deficient	5	100.0%	7	100.0%	-	14	100.0%	20	100.0%	-	0	0.0%	2	100.0%	-
	normal	0	0.0%	0	0.0%		0	0.0%	0	0.0%		0	0.0%	0	0.0%	

*For Vitamin K, the dietary reference intake is Adequate intake (AI) rather than recommended daily allowance (RDA).

There was no significant difference in micronutrient consumption within the study subjects when analyzed according to age and gender (Table 5).

### Food intolerance

**Fig 1:**
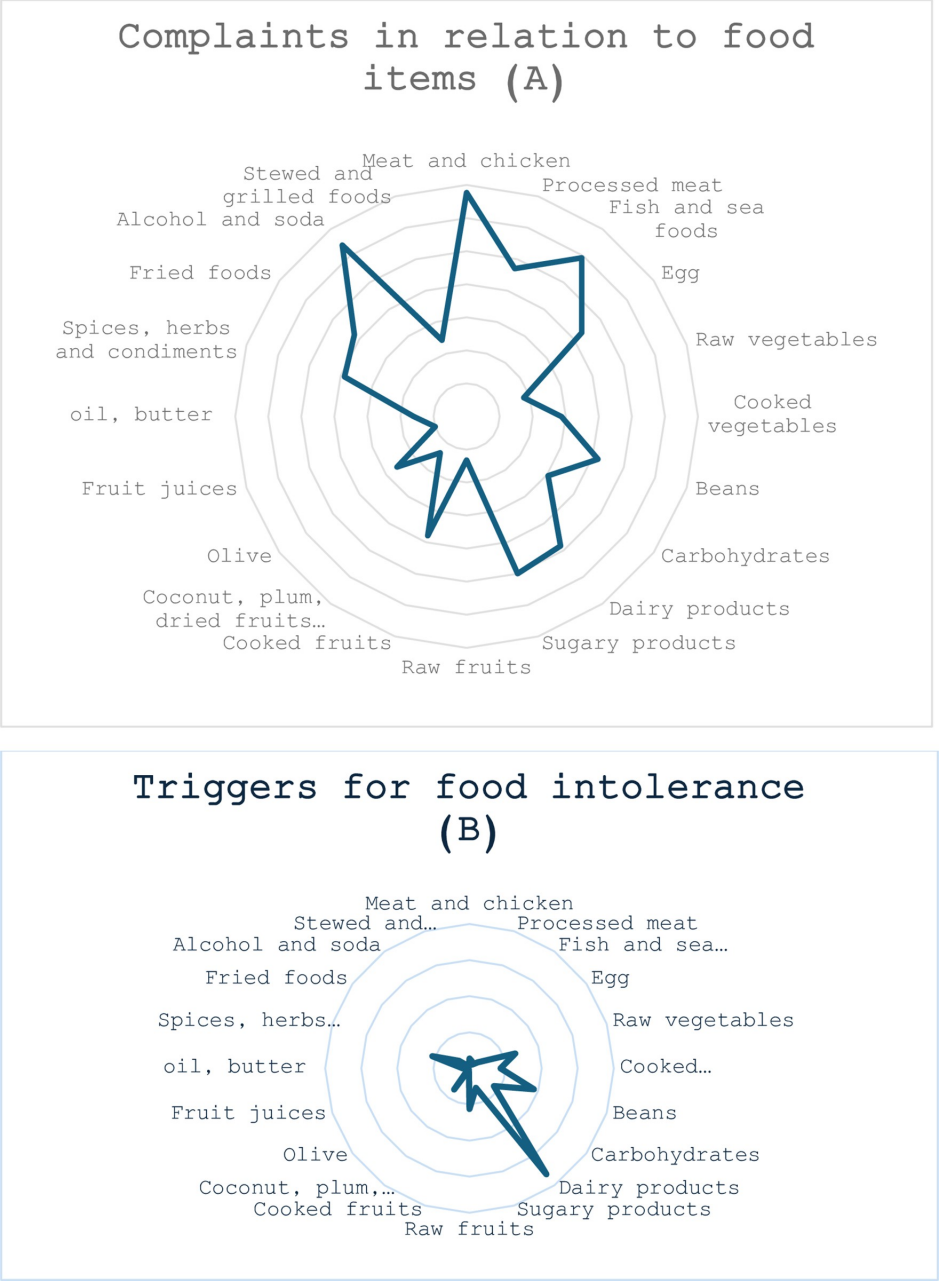
(A) Complaints regarding food items and (B) triggers for food intolerance among patients with CIBD.

Complaints about some food items and common triggers for intolerance in CIBD patients are shown in Fig 1. Patients with CIBD were complaining about wide ranges of food items (Fig 1A). On the other hand, food intolerance was extensively seen with dairy products, high-fiber foods (raw vegetables and beans) and spicy foods (Fig 1B).

### Bivariate analysis

A significantly proportion of patients identified with malnutrition followed specific nutrition regimen outside the flare, had a nutritional follow up, were currently taking corticosteroids and had a severe form of the disease compared to patients who were well nourished (Table 6). It is of note that logistic regression did not reveal any significant association among studied factors and the malnutrition status.

**Table 6:** Factors associated with risk of malnutrition among patients with CIBD.

		Malnutrition				
		Well nourished		Malnourished		p
		N	%	N	%	
Diet out of flare	no	33	82.5	3	37.5	**.007**
	yes	7	17.5	5	62.5	
Nutritional follow up	no	39	97.5	4	50.0	**< .001**
	yes	1	2.5	4	50.0	
Current treatment: corticosteroids	no	33	82.5	3	37.5	**.007**
	yes	7	17.5	5	62.5	
Severity	Mild	27	67.5	2	25.0	.013
	Moderate	7	17.5	1	12.5	
	Severe	6	15.0	5	62.5	

Bolded numbers denote statistically significant p-values.

## Discussion

In the patients with CIBD in this study, there was a high prevalence of food intolerance, leading to difficulties in consuming a variety of foods. This resulted in limited dietary options and nutrient deficiencies. Additionally, due to the restrictive nature of their diets, CIBD patients have low usage of dietary supplements, further exacerbating the risk of malnutrition. The combination of food intolerance, low dietary supplement use, and the chronic inflammation associated with CIBD can contribute to a higher prevalence of malnutrition in these patients [22].

In Table 2 it was noticed that malnutrition occurred (17%) in the CIBD diagnosed subjects, more specifically our results were obtained as follows: 10.4% for mild-moderate undernutrition and 6.3% for severe undernutrition. These findings have been consistent with a previous investigation who assessed malnutrition among 107 Australian CIBD outpatients and consequently found a similar malnutrition rate of 16.3% for UC and CD patients by using the SGA (subjective global assessment) tool [23]. However, a Canadian multicentered study including 249 CIBD patients, classified 23.3% of its sample as malnourished also by using SGA [24]. However, other studies in literature regarding CIBD have used different tools for malnutrition assessment therefore this could be limiting for comparison between results.

For example, a single-center cohort study observed elevated occurrence (>30%) of malnutrition in individuals with CIBD but the assessment was made by using the criteria set by the European Society for Clinical Nutrition and Metabolism [25]. In addition, after multiple studies, methods that were used were found affected by different BMI scores [24], QOL [23] or even vitamins levels [25, 26] and biochemical parameters [27] leading to inconstancy in undernutrition results varying from 16% to 82.8%.

Most early studies as well as current work focus on showing that self-reporting information concerning CIBD patients before and after flares are valuable for several reasons: first, and most importantly, it can help in the understanding of dietary triggers and patterns that contributes to the CIBD flares [28, 29]: by tracking food intake, patients can make connections between specific foods and symptoms exacerbation in order to avoid the consumption of inadequate foods. Second, in terms of treatment planning, self-reporting data before and after flares is highly beneficial for physicians and dietitians in order to build a personalized strategy for optimal recovery for every patient [30]. Moreover, self-reported dietary data can contribute to research on the triggers and patterns of food intake. These can be used to identify trends, common triggers and raise awareness against dietary habits that could be harmful for all CIBD patients [31]. In our study, planned comparison between dietary habits amid and after the CIBD flares revealed interesting results regarding patients’ behaviors. For example, in Table 3, we can identify that only 14.6% of CIBD participants use supplementation as part of their diet and only 10.4% consume vitamins’ supplementation (Vit D and Vit E). Whereas, during remission period, none of the patients have been found to use dietary supplements. Our results suggest that after a CIBD flare, vitamins supplementation consumption is not resumed, or they are not even part of dietary routine of CIBD patients. A previous study [32] explained that CIBD patients are more vulnerable to nutrient deficiencies, therefore regular nutritional assessment is advised to optimize nutritional parameters. On top of that, after a flare, patients would undergo a supplementation course at the onset of therapy that’s why supplementation rate increases in the remission episodes [33].

In addition, in Table 4, we can identify that the rate of adherence to diet prescription amid flares (50%) is higher than that during the remission episode (25%). Amid flares, 29.2% of patients start peeling fruits and vegetables that they consume whereas in the remission period only 2.1% of patients would peel their fruits and vegetables. This suggests that patients may try to change their dietary habits, possibly by giving more attention to their food consumption during the periods of flare. Moreover, amid flares, 25% of patients will stop eating fatty foods and snacks compared to only 2.1% of patients who will stop eating these foods during remission. In fact, we can understand that during CIBD flares, patients are being more careful regarding their dietary intake by reducing their consumption of fatty foods because foods high in saturated fats can contribute to CIBD flares [32, 33]. During a CIBD flare, 12.5% of patients stop consuming sugary foods and snacks compared to only 10.4% who opt to abstain during periods of no flares. The results tie well with previous studies wherein we noticed that CIBD patients often modify the diet, typically by avoiding fatty, fried foods, fruits and vegetables and sugars during flares to avoid further exacerbations [34, 35].

Finally, we can even note that sources of dietary advice may vary between different periods of the disease. For example, amid flares dietary advice may come from physicians by 33.3%, dietitians by 2.1% and personal belief by 14.6%. Whereas outside flares, referring to a healthcare specialist have been less reported by CIBD patients: 16.7% would consult a physician and 75% would not take dietary advice. Nowadays, a surge of studies [36–38] have been promoting the need for educational and structural interventions by healthcare professionals in order to assess and treat CIBD, thereby preventing flares and sustaining patients’ well-being.

Data demonstrates that individuals suffering from malnutrition were more likely to follow a diet beyond the flare with a rate of 62.5% compared to those who are well-nourished where only 17.5% had to modify their diet. To substantiate our findings, a positive correlation between being malnourished as a CIBD patient and having to undergo a diet out of flare has been obtained by Pearson Chi-square test (p = 0.007). Similarly, a previous study regarding CIBD management summarized major guidelines of the ESPEN (European Society for Parenteral and Enteral Nutrition): [39] In accordance with our findings, one of the recommendation was that “ONS (Oral Nutritional Supplements) or EN (enteral nutrition) can be recommended in patients with CD in remission, if undernutrition cannot be treated sufficiently by dietary counselling”. It has been strongly consented that malnourished patients who are chronically ill have to undergo strict diets as EN/ONS after a flare [40]. In fact, a diet out of flare have been extensively researched over the years and have been found helpful with mucosal healing and enhancing barrier defense. Second, our findings present malnourished patients undergo a nutritional follow up by a rate of 50% among the total sample. This has been supported by many studies and recommendations stating that adequate dietary habits can avoid CIBD relapse and adverse effects. a literature review [41] explained that a restrictive diet could lead to drastic vitamin deficiencies which can aggravate the patients’ state: that’s why a professional intervention must encourage a healthy and balanced diet instead of extreme restrictions. Based on the ESPEN guidelines [39], it has been stated that seeking consultations from a dietitian is a key component of the holistic approach recommended for CIBD patients in order to avoid malnutrition and nutrition-related disorders. Third, a significantly larger fraction of patients with malnutrition undergoes a treatment of corticosteroids with a rate of 62.5% compared to those who were well-nourished. Based on the Pearson Chi-Square Tests, we found a positive correlation between being malnourished and the need to have a treatment of corticosteroids with p = 0.007. In fact, it has been discussed that nutritional impairment can influence immune cell populations and function which makes the immune system unable to fight inflammation and repair tissues in the gut [42, 43]. Therefore, a course of corticosteroids is recommended for active CIBD to induce remission and alleviate uncontrolled inflammation due to malnutrition [44]. Finally, the highest rate equal to 62.5% regarding severity of CIBD corresponded to malnourished patients who had severe CIBD symptoms. In our analysis, malnutrition, and severity of CIBD have been found positively correlated with a p-value equal to 0.013. Furthermore, a recent study [21] explained that malnourished CIBD patients are advised to be screened annually due to higher risks for adverse outcomes (venous thromboembolism, longer hospital admission and increased mortality). These results tie well with a previous study [45] showing that malnutrition was prominently observed among CIBD admissions and it reflected a more severe underlying disease that would lead to detrimental outcomes. In Table 6, we see that 50% of malnourished patients were receiving nutritional follow-up while only 2.5% of well-nourished patients had a dietary follow-up. This suggests that a higher proportion of malnourished patients were receiving nutritional follow-up compared to well-nourished patients. This indicates that the clinicians are more likely to provide care and support for those who are malnourished due to higher health related risks in this population. We must note that there is a potential gap in care, and a small portion of well-nourished are receiving nutritional consultations, which suggests more dietary attention to all patients regardless of their nutritional status.

### Limitations

This study has several potential limitations. It has limited its population to an adult population and our small sample may not be considered representative of the population. While CIBD can also impact children as well as adults. For instance, a previous study has recorded that 1 out of 1299 children [46] between the age of 2 to 17 is affected by CIBD. We should also highlight the fact that the count of elderly patients (>65 years old) with CIBD was significantly low. Therefore, having limited age groups puts our study at risk for lack of precision in the results. Moreover, the cross-sectional nature of the study hinders the generation of a causal relationship between type of food intake and the increased disease activity. This small sample size made it more difficult to obtain multiple subgroups which led to obstacles in conducting multiple comparison and the single-center sample of the study taken from 1 hospital (Al-Sahel Lebanese Hospital) somewhat reflects similar treatment patterns and maybe similar socioeconomic status of the participants which leads to more bias in our study. We can mention that the gender distribution is not equal with 60.9% females and 39.1% males, this can lead to bias in our results since women and men follow different dietary patterns and behaviors regarding food intake [47, 48]. The methods used for body composition tests could show error in the measurements’ results if the clinician was not skilled and unfamiliar with the equipment. In addition, this bivariate study examined only two variables, hence its capacity to provide a profound comprehension of the objectives was limited. On top of that, the use of subjective questionnaires and relying on self-assessment in the course of this investigation has introduce bias in our results and decrease the precision of our analysis [47].

## Conclusion

In summary, our study has identified uncharted areas of malnutrition related to CIBD. In line with other previous studies, our investigation concluded that the prevalence of malnutrition is 16% among CIBD patients. We have found a positive association between malnourished CIBD patients and the fact that they would follow a diet out of flare, to undergo a nutritional follow up and to go through a treatment of corticosteroids. This study has contributed valuable insights into the understanding that some food items could be associated to periods of increased disease activity in CIBD patients and that awareness/intervention regarding nutrition must be provided by healthcare professionals (dietitians, physicians…) to decrease the need for second line therapy. In addition, this self-reported food intolerance paper gives an insight for patients on food items usually avoided by CIBD patients during flares. Future research should consider the causal association between food types and the decrease or increase in CIBD activity.
