# Correction: Self-reported food intolerance, dietary supplement use and malnutrition in chronic inflammatory bowel diseases: Findings from a cross-sectional study in Lebanon

**DOI:** 10.1371/journal.pone.0317159

**Published:** 2025-01-02

**Authors:** Maha Hoteit, Nour Ftouni, Malak Olayan, Souheil Hallit, Joya Maria Karam, Mahmoud Hallal, Samer Hotayt, Bilal Hotayt

There is an error in affiliation 1 for author Maha Hoteit. The correct affiliation 1 is: Food Science Unit, National Council for Scientific Research of Lebanon, Beirut, Lebanon (CNRS-L).
